# IL-1β Promotes a New Function of DNase I as a Transcription Factor for the Fas Receptor Gene

**DOI:** 10.3389/fcell.2018.00007

**Published:** 2018-02-06

**Authors:** Dhivya Thiyagarajan, Hege L. Pedersen, Natalya Seredkina, Kjersti D. Horvei, Lorena Arranz, Ramon Sonneveld, Tom Nijenhuis, Johan van der Vlag, Ole P. Rekvig

**Affiliations:** ^1^RNA and Molecular Pathology Research Group, Department of Medical Biology, University Hospital of North Norway, Tromsø, Norway; ^2^Stem Cell Aging and Cancer Research Group, Department of Medical Biology, Faculty of Health Sciences, UiT-The Arctic University of Norway, Tromsø, Norway; ^3^Department of Nephrology, Radboud University Medical Center, Nijmegen, Netherlands; ^4^Department of Radiology, University Hospital of North Norway, Tromsø, Norway

**Keywords:** DNase I, Fas receptor, transcription factors, IL1beta, lupus nephritis

## Abstract

Recently we described that endonuclease inactive DNase I translocated into the nucleus in response to increased endogenous IL-1β expression. Here, we demonstrate impact and function of translocated DNase I in tubular cells. Effect of cytokines on expression level and nuclear localisation of DNase I and corresponding levels of Fas receptor (FasR) and IL-1β were determined by confocal microscopy, qPCR and western blot analyses, in presence or absence of siRNA against IL-1β and DNase I mRNA. Nuclear DNase I bound to the *FAS* promotor region as determined by chromatin immuno-precipitation analysis. Data demonstrate that; (i) translocation of DNase I depended on endogenous *de novo*-expressed IL-1β, (ii) nuclear DNase I bound *FAS* DNA, (iii) FasR expression increased after translocation of DNase I, (iv) interaction of *exogenous* Fas ligand (FasL) with upregulated FasR induced apoptosis in human tubular cells stimulated with TNFα. Thus, translocated DNase I most probably binds the promoter region of the *FAS* gene and function as a transcription factor for FasR. In conclusion, DNase I not only executes chromatin degradation *during* apoptosis and necrosis, but also primes the cells *for* apoptosis by enhancing FasR expression.

## Introduction

Chromatin fragments play a decisive role in immune complex formation with anti-double stranded DNA (dsDNA) antibodies in lupus nephritis. Such immune complexes are detected in glomeruli as electron dense structures (EDS) by electron microscopy, or as granula in glomerular membranes and matrices by immunofluorescence assays (Berden et al., 1999; Kalaaji et al., 2006; Mortensen and Rekvig, 2009; van der Vlag and Berden, 2011; Seredkina et al., 2013). As lupus nephritis progresses from early to advanced stages, we observed in our studies a distinct increase of chromatin deposits in glomerular basement membranes (GBM), whereas deposits in early nephritis were confined to the mesangial matrix. Correlating significantly in time with chromatin accumulation, we found silencing of the gene encoding renal DNase I (Fenton et al., 2009; Seredkina et al., 2009; Seredkina and Rekvig, 2011) that accounts for more than 80% of total renal endonuclease activity (Basnakian et al., 2005; Zykova et al., 2008). This expression pattern of DNase I is observed consistently in murine and human lupus nephritis (reviewed in Seredkina et al., 2013). These studies point out renal DNase I as a protective factor when it is expressed, whereas *DNASE1* gene silencing is related to progression of the disease (Zykova et al., 2008; Fenton et al., 2009; Seredkina et al., 2009).

The DNase I endonuclease was described already in 1946 by McCarthy et al. (McCarty, 1946). Despite knowing the enzyme for seven decades, we still do not understand regulation of DNase I expression and activity, its dynamic subcellular migration, and localization (Choi et al., 2008), nor its role in apoptosis and necrosis (Samejima and Earnshaw, 2005; Kawane and Nagata, 2008), particularly in context of autoimmunity (Napirei et al., 2000; Martinez-Valle et al., 2009). During a longitudinal study on expression profiles in (NZBxNZW)F1 (BW) mice, we observed a tendency for DNase I up-regulation during mesangial nephritis, before a subsequent and distinct down-regulation of the gene during progressive disease (Fenton et al., 2009; Seredkina and Rekvig, 2011). In addition, we have observed nuclear localization of renal DNase I in tubular cells in human lupus nephritis (Thiyagarajan et al., 2015). Studies on cultured human renal proximal tubular epithelial cells (RPTEC) have also shown translocation of DNase I into the nucleus under certain conditions (Thiyagarajan et al., 2015).

Detailed analyses of DNase I expression by Western blot, gel zymography, and mass spectrometry (MS) revealed three major variants of the DNase I protein in resting tubular cells. Two DNase I variants were determined to be products of the *DNASE1* gene by mass spectrometry (MS) and were expressed in the cytoplasm; one enzymatically active with molecular weight (MW) of 40 kDa, and another enzymatically inactive with MW around 55 kDa. The DNase I variant that translocated into the nucleus was enzymatically inactive and differed from the other detected DNase I molecules, as it had a MW of 52 kDa (Thiyagarajan et al., 2015). Studies from Oliveri et al. suggested that recombinant DNase I added to cell cultures could act as transcriptional factor for FAS and increase the expression of cell surface fas receptor (FasR) in human cells (Oliveri et al., 2004). This encouraged us to analyse if nuclear DNase I acts as a transcription factor for *FAS* in RPTEC.

The present study aims to describe three phenomena linked to renal *DNASE1* gene expression and subcellular compartmentation in response to different stimuli. First, since the *DNASE1* gene tended to be upregulated in mesangial nephritis (Fenton et al., 2009; Thiyagarajan et al., 2015), we assumed that pro-inflammatory cytokines may be responsible for this phenomenon. Second, the same pro-inflammatory cytokines were expected to induce nuclear translocation of DNase I; and third, nuclear translocated DNase I presumably had another biological significance than to promote chromatin degradation. This consideration is based on the fact that translocated DNase I was demonstrated to be endonucleolytically inactive (Thiyagarajan et al., 2015). The results presented here confirm the rationale of this threefold idea and describe a novel role of DNase I as a transcription factor regulating FasR expression. These findings imply that DNase I has a biological role as a pro-apoptotic protein that prime the cells for apoptosis, whereas it also contributes as an endonuclease executing chromatin degradation in context of apoptotic and necrotic cell death.

## Materials and methods

### Ethic statement

The mouse study was approved by The National Animal Research Authority (NARA) (approval ID: 07/11167, ID-178).

### Murine tissue samples

Female BW and female age-matched BALB/c mice were purchased from Jackson Laboratory, Bar Harbor, Main, USA. Kidney tissue was collected as described (Fenton et al., 2009), and BW mice were grouped according to stages of murine lupus nephritis. Group 1 pre-nephritic mice (no glomerular deposits of chromatin or IgG), Group 2 mice with mesangial nephritis (mesangial deposits of chromatin-IgG complexes), and Group 3 mice with end-stage disease (deposits of chromatin-IgG complexes in mesangium and in GBM, Fenton et al., 2009).

### Proteins and antibodies

The recombinant proteins used in this study were: human TNFα (210-TA) and IL-1β (201-LB 1) from R&D system (Minneapolis, MN, USA), and Fas ligand (FasL) protein (ab109359) from Abcam (Cambridge, UK). Caspase 3 control cell extracts (untreated and cytochrome c treated Jurkat cells) were obtained from Cell Signaling Technology (Boston, USA). Alexa Fluor® 488 annexin V was purchased from Life Technologies (California, USA). Kineret (anakinra)—IL-1 receptor antagonist (IL-1Ra) was obtained from SOBI, Sweden.

The following antibodies were used in this study: rabbit anti-DNase I (ab113241, Abcam-Cambridge, UK), goat anti-Trap1(sc-69289), and rabbit anti-DNase I (sc30058) from (Santa Cruz, Texas 75220 U.S.A), rabbit anti-DNase I (LS-B4846), rabbit anti-FasR (LS-C152529, SC-74540), rabbit anti-FasL antibody (LS-C176019), mouse anti-FasL antibody (LS-C35867), and mouse anti-caspase 3 antibody (LS-B2125) were from Lifespan Biosciences (Seattle, WA). Cleaved caspase 3-specific antibody (9661) were from Cell Signaling Technology (Europe, B.V.), rabbit anti-actin antibody (A2066) from Sigma-Aldrich (St. Louis, MO) and mouse IL-1 beta /IL-1F2 Antibody (AF-401-NA) from R&D System. Alexa 595-conjugated anti-rabbit IgG, Alexa 488-conjugated anti-goat IgG, DAPI and Alexa 488-conjugated anti-mouse IgG secondary antibodies were all from Invitrogen (California, USA). Alexa Fluor® 488 annexin V was purchased from Life Technologies (California, USA), HRP-conjugated anti-rabbit IgG were from Invitrogen (California, USA), and HRP-conjugated anti-mouse IgG were purchased from Dako (Oslo, Norway).

### Cell culture experiments

Primary human renal proximal tubule epithelial cells (RPTEC) (Clontec, Lonza, Basel, Switzerland) have recently been validated for the present experiment (Thiyagarajan et al., 2015). The cells were grown in Clontec REGM™ BulletKit (CC-3190) containing Renal Epithelial Cell Basal Medium with the following growth supplements: hEGF, Hydrocortisone, Epinephrine, Insulin, Triiodothyronine, Transferrin, GA-1000, and fetal bovine serum, at 37°C in 95% humidified air and 5% CO_2_. The cells were grown to 80% confluence (without growth arrest) and stimulated with TNFα (0, 20 ng/ml) or IL-1β (0, 2.5 ng/ml), if not otherwise stated in the text. These concentrations were based on results from serial-dilution experiments as demonstrated below. The cells were harvested at 24 and 48 h for mRNA analysis by qPCR and for protein expression analysis by Western blot and confocal microscopy. All cell stimulation experiments were performed in triplicates and repeated at least three times.

### Short interference RNA (siRNA) transfection

In this study, 2 × 10^5^ cells/well in six-well dishes and 2 × 10^4^ cells/well in confocal plates were seeded 24 h before transfection. After 24 h the media was removed and transfection was proceeded with fresh media containing 20 ng/ml TNFα without and with 20 nM scrambled smart pool DNase I siRNA or IL-1β siRNA (Dharmacon) and negative siRNA control (Sigma) using Lipofectamine 2000 (Invitrogen). The cells were harvested after 48 h after transfection. siRNA against P62 was used as a positive control for transfection in separate wells.

### RPTEC stimulation with IL-1β in presence of IL-1 receptor antagonist

RPTEC were grown to 80% confluence and cells were stimulated with 2.5 ng/ml of IL-1β alone or together with 200 ng/ml of IL-1 receptor antagonist (IL-1Ra). Cells were harvested after 0-6-12-24-42-48 h and analyzed by qPCR.

### ELISA

RPTEC were grown to 80% confluence and cells were stimulated with 20 ng/ml of TNFα for 48 h. Cell supernatant and cell lysate from both unstimulated and TNFα stimulated cells were analyzed for IL-1β using IL-1β Quantikine ELISA kit from R&D systems. Quantitative determination of human IL-1β in both cell supernatant and cell lysate were performed according to the instruction by the manufactures. Experiments were run in triplicates and results were represented from two individual experiment.

### Stimulation of RPTEC with TNFα and subsequent incubation with fas ligand

For the FasL experiment, RPTEC were grown to 80% confluence and cells were stimulated with 20 ng/ml of TNFα for 48 h. After 48 h of stimulation, RPTEC were incubated with various concentrations of FasL protein (0, 25, 125 ng/ml) and grown for 48 h. Thereafter the cells were harvested and stored at −80°C. The apoptotic cells were analyzed and counted using TUNEL assay and Annexin V staining.

### RNA isolation and cDNA synthesis

Total RNA was isolated from RPTEC using TRIzol ® (Invitrogen, CA, US), as described by the manufacturer. RNA quality and concentration were determined spectrophotometrically by Nano Drop (Nano Drop technologies, Wilmington, USA). RNA samples were reverse-transcribed with random primers using High Capacity cDNA Reverse Transcription kit (Applied Biosystems, Foster City, USA).

### Gene expression analyses

Quantitative real time PCR (qPCR) was performed using ABI Prism 7900HT Sequence Detection System (Applied Biosystems). Pre-designed FAM-labeled gene expression assays (Applied Biosystems) were purchased and accession numbers for human analyses are given in Table 1. TATA binding protein (TBP) was used as endogenous control. The relative expression levels were calculated using the ddCT method.

**Table 1:** Primer-probes used in this study, and their accession number.

**Primer-Probes**	**Accession number**
DNaseI	Hs00173736_m1
TRAP1	Hs00212476_m1
TBP (house-keeping gene)	Hs00427621_m1
FASR	Hs00236330_m1
Caspase-3	Hs00234385_m1
Caspase-7	Hs0016152_m1
Caspase-9	Hs00154261_m1
Bcl2	Hs00608023_m1
IL-1beta	Hs01555410_m1
FASL	Hs00181225_m1
P62	Hs00177654_m1

### Western blot

For Western blot analysis, cells were harvested using LDS buffer (Invitrogen) and the protein concentration was determined using protein quantification assay from Macherey-Nagel (Duren, Germany) according to the manufacturer instruction. Approximately 10 μg protein per well were loaded onto a 4–12% Nu PAGE Bis-Tris gel (Invitrogen). SDS-PAGE and Western blotting were performed according to standard procedures as described in detail (Kalaaji et al., 2006) and actin was used as a loading control.

### Confocal microscopy

RPTEC were seeded in a special 8 μ well-slide (IB80826) from Ibidi (Munich, Germany). The cells were fixed in 4% paraformaldehyde (KEBO, Oslo, Norway) in 1xPBS and incubated for 10 min on ice. The cells were permeabilized using 0.1% Triton X-100 (VWR International Oslo, Norway) in PBS for 5 min, followed by blocking in 3% BSA for 30 min and incubation with the appropriate primary and secondary antibodies for 60 and 30 min, respectively. Protein localization was detected using Zeiss-LSM510 Meta confocal microscopy (Oberkochen, Germany).

### Immunohistochemistry (IHC) analyses of murine kidneys

Immunohistochemical (IHC) staining of DNase I was performed as described and Polink-2 Plus HRP with DAB kit (Newmarket Scientific, UK) was used as detection system. IHC staining of IL-1β was performed on frozen kidney sections as previously described (Hedberg et al., 2013).

### Chromatin immuno-precipitation (ChIP)

The chromatin immuno-precipitation (ChIP) experiment was performed using anti-DNase I antibody as the assay-specific antibody, or non-specific rabbit IgG immunoglobulins as a negative control. Sheared chromatin and ChIP using anti-histone H3 antibodies were included as positive controls. The gene to which DNase I was bound was identified by qPCR. The ChIP assay was performed using unstimulated RPTEC or RPTEC stimulated with 20 ng/ml of TNFα. Cells were harvested 48 h after stimulation, and a Magna ChIP A assay (Merck Millipore, MA, USA) was performed according to the manufacturer's protocol. Briefly, proteins were cross-linked to the DNA with formaldehyde, cells were lysed, and samples were sonicated twice for 30 s on ice at a 22-mm amplitude using a Soniprep 150 (MSE, London, UK). Samples were incubated with 5.0 μg of rabbit polyclonal anti-DNase I antibody (ab113241 Abcam Inc., MA, USA) and with 5.0 μg of rabbit polyclonal anti-DNase I antibody (sc30058) with appropriate controls. Immuno-precipitates were enriched with protein A magnetic beads. Subsequently, chromatin complexes were eluted, the crosslinks were reversed, and DNA was isolated. The presence of FasR DNA was evaluated using qPCR with primers corresponding to the FasR promoter region (5′ GGGTCTTCCTCATGGCACTAAC 3′ and 3′CTCCTGAGGGCTTTCCATCAC 5′). Thereafter, samples were loaded on a 2% agarose gel and visualized using ProXima C16 software version 3.0 (Isogen Life Science, De Meern, The Netherlands).

### Annexin V staining

Annexin V staining was performed according to the manufacturer's instructions. In short, unstimulated, TNFα-stimulated or TNFα-stimulated in presence of FasL, adherent and non-adherent RPTEC were washed in cold phosphate-buffered saline (PBS). Washed non-adherent cells were centrifuged, the supernatant discarded, and the cell-pellet was resuspended in annexin-binding buffer (10 mM HEPES, 140 mM NaCl, 2.5 mM CaCl_2_, pH 7.4). Adherent and non-adherent RPTEC were then diluted to a cell density of about ~1 × 10^6^ cells/ml. In situations where cell death was marginal, all non-adherent RPTEC were pelleted and subjected to analyses. Five microliters of Alexa Fluor® 488 annexin V were added to each 100 μl of cell suspension before the cells were incubated at room temperature for 15 min. Next, the cells were subjected to DAPI for 1 min and washed with annexin-binding buffer. The non-adherent cells were placed on a slide. Both the non-adherent and adherent annexin V and DAPI stained RPTEC were subjected to a Carl Zeiss LSM 700 confocal microscopy for fluorescence detection.

### Terminal deoxynucleotidyl transferase dUTP nick end labeling (TUNEL) assay

The terminal deoxynucleotidyl transferase dUTP nick end labeling (TUNEL) kit was purchased from Roche **(**Indiana, USA) and the assay was performed according to the manufacture's instruction for both non-adherent and adherent cells. Briefly, unstimulated, TNFα stimulated and TNFα stimulated and FasL treated RPTEC cells were washed before the adherent cells were fixed in 4% paraformaldehyde (PFA) in PBS for 10 min on ice. The non-adherent cells were fixed with 2% PFA for 60 min in room temperature prior to centrifugation and removal of supernatant. The cells were permeabilized using 0.1% Triton X-100 (VWR International) in PBS for 5 min. The cells were subsequently treated with TUNEL Mix containing TUNEL label (11767291910; Roche) and the terminal deoxynucleotidyl transferase enzyme (11767305001; Roche), and incubated at 37°C for 1 h. The cells were washed with PBS and nuclei were stained by DAPI and subsequently subjected to confocal microscopy using the Carl Zeiss LSM 700 confocal microscopy. As a positive control in the TUNEL assay, RPTEC were exposed to 10U recombinant DNase I to induce single-stranded (TUNEL-positive) nicks, and the negative control was represented by TUNEL in absence of terminal deoxynucleotidyl transferase.

### Caspase GLO 3/7 assay

RPTEC were treated with 20 ng/ml of TNFα with or without 125g of FasL for 48 h. Following treatments, caspase 3/7 activity were measured using the Caspase-Glo 3/7 assay kit (Promega, Madison USA). Briefly, plates with cells were removed from the incubator and allowed to equilibrate to room temperature for 30 min. Caspase-Glo reagent (100 μl) was added to each well before it was gently mixed with a plate shaker at 300–500 rpm for 30 s. The cells were then incubated at room temperature for 30 min−1 h. The luminescence of each sample wasmeasured in a Clariostar microplate reader (BMG-Labtech, Ortenberg, Germany). The experiments were performed in triplicate and repeated in three independent experiments.

### Statistics

Graph pad Prism was used to calculate the significance and the data are presented as mean of three parallels (± *SD*). An unpaired *t*-test was performed to test differences between the cytokine stimulated/siRNA transfected and non-transfected cells and for ELISA. One-way ANOVA with Dunett *post-hoc* test was performed at different concentrations of cytokines to non-stimulated cells and for cas3/7 GLO Assay. For each parameter; *p* < 0.05 was considered significant and represented as ^*^. If *P*-value is ≤ 0.05 represented as ^*^ ≤ 0.005 as ^**^ and *P*-value ≤ 0.0005 as ^***^. All observations were included and Spearman was used for significance testing.

Fuji Image J program was used to measure the nuclear staining intensity for all the confocal images.

## Results

### Stimulation of RPTEC with TNFα promotes nuclear translocation of the DNase I protein and increased expression levels of DNase I, IL-1β, and FasR

**Figure 1:**
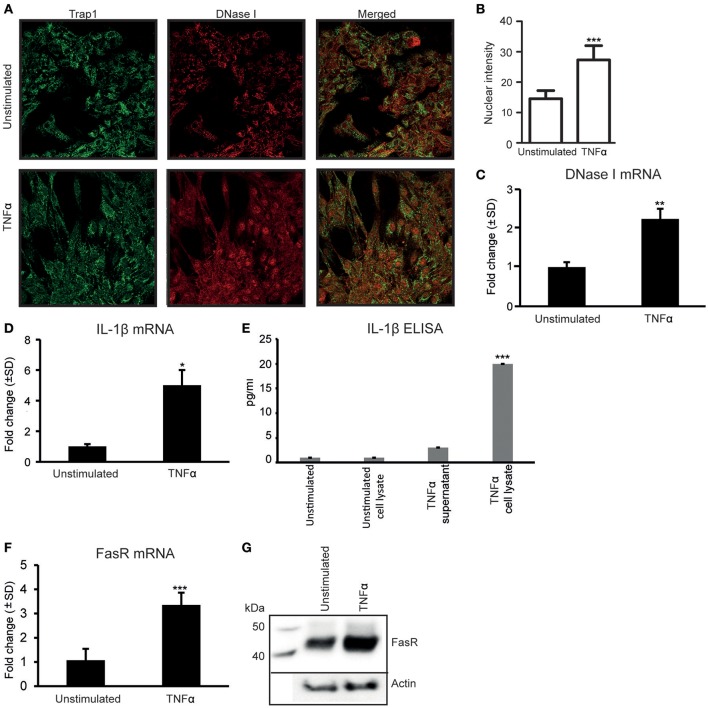
Stimulation of human renal proximal tubular epithelial cells (RPTEC) with TNFα enhances DNase I, FasR, and IL-1β expression levels and induces translocation of the DNase I protein into the nucleus. Stimulation of RPTEC with 20 ng/ml of TNFα for 48 h enhances DNase I expression and induces translocation of the DNase I protein into the nucleus. Trap1 staining was used as a cytoplasmic marker. Upper panel: unstimulated RPTEC; lower panel: TNFα stimulated RPTEC (DNase I: red, Trap 1: green) **(A)**. Intensity of nuclear DNase I staining was measured using ImageJ **(B)**. TNFα stimulation enhances mRNA expression level of DNase I **(C)**. In addition, the mRNA expression levels and protein expression (ELISA) levels of IL-1β, represented from two individual experiments (**D,E**, respectively), were increased. At the same time, expression levels of FasR mRNA and protein level were significantly upregulated in TNFα-stimulated cells (**F, G**, respectively). Significances: ^*^P < 0.05; ^***^P < 0.0005.

As we have previously demonstrated (Thiyagarajan et al., 2015), stimulation of RPTEC with 20 ng/ml of TNFα *in vitro* for 48 h upregulates DNase I protein (Figure 1A). Nuclear intensity of DNase I after TNFα stimulation shows a significantly higher staining in the nuclei of TNFα stimulated cells (Figure 1B). Stimulation of RPTEC with 20 ng/ml of TNFα significantly upregulates DNase I mRNA (Figure 1C). Also, TNFα upregulates IL-1β mRNA levels (Figure 1D) and IL-1β protein levels (Figure 1E) and translocates DNase I into the nucleus (Figure 1A, Trap1 (green) is used as a cytoplasmic marker). Because TNFα is a potent inducer of apoptosis (Krown et al., 1996; He and Ting, 2002), the nuclear translocation of DNase I in response to TNFα is probably linked to an apoptotic process. Particularly, the FasR mRNA (Figure 1F) and the FasR protein expression levels (Figure 1G) were significantly upregulated in TNFα-stimulated cells compared with unstimulated cells. As shown in Supplementary Figure 1A, TNFα-stimulation of RPTEC induced *FAS* gene expression in a dose-depended manner. While FasR mRNA and FasR protein were constantly expressed in unstimulated RPTEC and increased upon TNFα-stimulation, FasL mRNA (qPCR, Supplementary Figure 1B) were undetectable after stimulation of the cells by TNFα or by IL-1β (Supplementary Figures 1B,C).

### Stimulation of RPTEC with IL-1β promotes nuclear translocation of DNase I and upregulation of FasR expression levels

**Figure 2:**
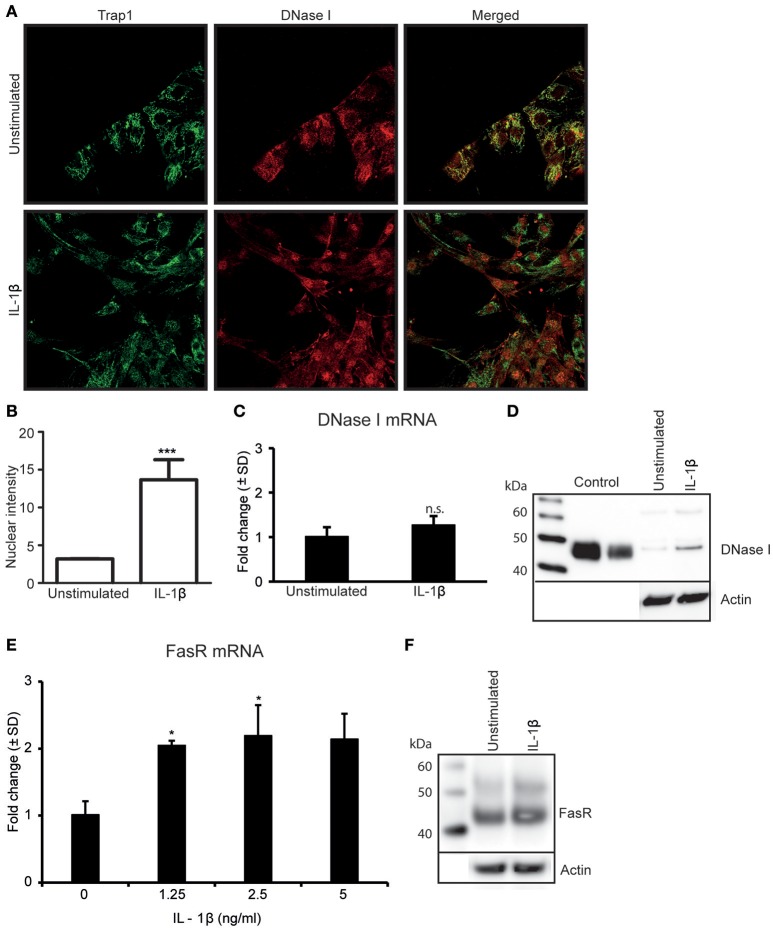
Stimulation of RPTEC with IL-1β promotes nuclear translocation of DNase I and upregulatesFasR expression levels. Stimulation of RPTEC with 2.5 ng/ml of IL-1β for 48 h did not upregulate DNase I protein expression but imposed nuclear translocation of DNase I as demonstrated by confocal microscopy (DNase I: red, Trap 1: green) **(A)**. Intensity of nuclear DNase I staining was measured using ImageJ **(B)**. Stimulation of RPTEC with 2.5 ng of IL-1β for 48 h did not affect DNase I mRNA **(C)** and protein expression levels **(D)**. Importantly, **e**nhanced FasR mRNA **(E)** and protein levels **(F)** were observed in cells after IL-1β stimulation. In this experiment, a nearby full FasR mRNA response was achieved when stimulating the RPTEC cultures with 1.25 ng/ml of IL-1β **(E)**. Significances: ^*^P ≤ 0.05; ^***^P ≤ 0.0005.

Since TNFα-stimulation of RPTEC upregulates IL-1β expression levels (Figures 1D,E) (Turner et al., 2007; Thiyagarajan et al., 2015), we analyzed whether IL-1β was involved in regulation of DNase I expression levels and/or its nuclear translocation. As shown in Figure 2A, IL-1β-stimulation did not significantly affect expression levels of DNase I *in situ* as demonstrated by confocal microscopy (Figure 2A). However, nuclear intensity measurements of DNase I after IL-1β stimulation show that IL-1β clearly promotes a nuclear translocation of DNase I (Figures 2A,B and Supplementary Figures 2A,B). Percentage of nuclear stained cells in stimulated vs. unstimulated cells were calculated and showed nuclear staining of almost 85% of stimulated cells compared to unstimulated cells (Supplementary Figure 2A). IL-1β did not affect the DNase I mRNA (Figure 2C), nor the DNase I protein expression levels significantly (Figure 2D). However, Importantly, IL-1β-stimulation induced a significant upregulation of FasR mRNA (Figure 2E) and FasR protein levels (Figure 2F), while it did not affect FasL expression levels in RPTEC (Supplementary Figures 1C). Interestingly, low doses of IL-1β were sufficient to induce upregulation of FasR mRNA levels (Figure 2E, Supplementary Figure 3A). Corresponding mRNA levels of IL-1β in response to different doses of IL-1β stimulation are demonstrated in Supplementary Figure 3B. This experiment indicates that as little as 0.037 ng/ml is sufficient to significantly upregulate both FasR and IL-1β mRNA levels above limits necessary for DNase I translocation. These data are important for understanding the results of experiments where TNFα-stimulation of RPTEC was intended to be inhibited with siRNA specific for IL-1β, or by addition of IL-1Ra (see below). Further studies are therefore needed to understand if endogenous, newly synthesized IL-1β is important for DNase I translocation (studies in progress).

### *De novo* synthesis of DNase I is not essential for nuclear translocation of DNase I nor for upregulation of FasR levels

**Figure 3:**
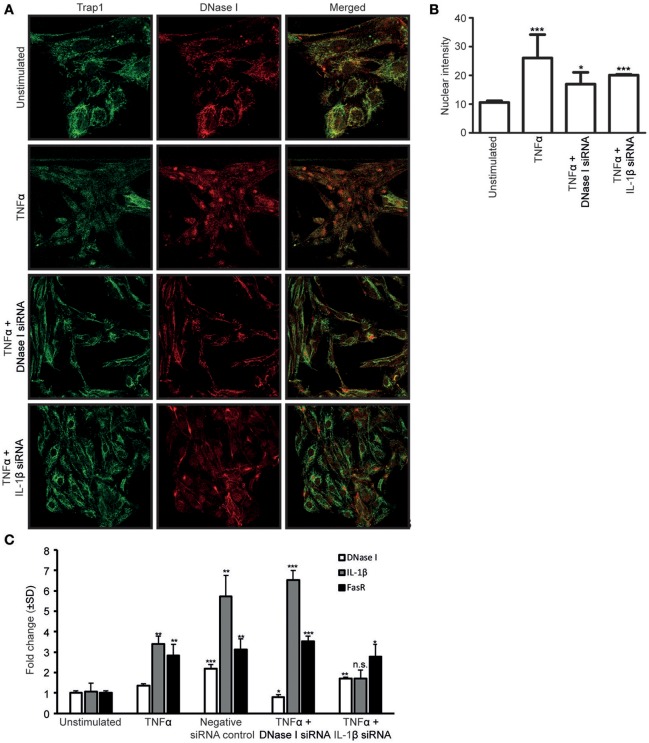
The siRNA against IL-1β nearby abolished the nuclear translocation of DNase I. Confocal microscopy analyses of DNase I (red) was performed on RPTEC stimulated with TNFα and simultaneously transfected with DNase I-specific or IL-1β-specific siRNAs. Trap1 staining was used as a cytoplasmic (green) marker. Unstimulated RPTEC present weak DNase I expression and no nuclear DNase I staining (**A**, first row). When the RPTEC were stimulated with TNFα, DNase I protein expression increased, and DNase I translocated into the nucleus (**A**, second row). Treating the cells with TNFα and transfecting with DNase I mRNA-specific siRNA, reduced the DNase I protein expression and nuclear DNase I translocation (**A**, third row). In addition, TNFα stimulation of IL-1β siRNA-transfected RPTEC reduced nuclear translocation of DNase I (**A**, fourth row). These results were confirmed with intensity measurements of nuclear DNase I staining **(B)**. DNase I, FasR and IL-1β mRNA levels were analyzed in RPTEC stimulated with TNFα. In addition, mRNA levels of RPTEC stimulated with TNFα and simultanously transfected with DNase I and IL-1β mRNA-specific siRNA are shown. As a control, RPTEC were treated with negative siRNA. **(C)**. TNFα-stimulation in the presence of DNase I-specific siRNA resulted in the reduction of DNase I mRNA level, while IL-1β and FasR mRNA levels were unaffected and remained the same as in TNFα-stimulation alone. In contrast, presence of IL-1β mRNA-specific siRNA in TNFα-stimulated RPTEC resulted in a significant reduction of FasR compared to TNFα stimulation alone, while DNase I gene expression level was unchanged. As expected, IL-1β mRNA expression level was reduced **(C)**. Significance: ^*^P ≤ 0.05; ^**^P ≤ 0.005; ^***^P ≤ 0.0005; ns *P* > 0.05.

To determine if newly synthesized DNase I was required for DNase I nuclear translocation, RPTEC were stimulated with TNFα and simultaneously transfected with DNase I-mRNA-specific or IL-1β-mRNA-specific siRNAs (Figure 3A). Furthermore, analyses were undertaken to determine if nuclear translocated DNase I is responsible for upregulation of the *FAS* gene. Thus, we investigated the links between TNFα, IL-1β, DNase I expression levels and its nuclear translocation and possible ability to enhance FasR expression levels.

Confocal microscopy analyses of DNase I (red) was performed on RPTEC after stimulation with TNFα and the effect of simultaneous transfection of the cells with DNase I mRNA-specific or IL-1β mRNA-specific siRNAs. Trap1 staining (green) was used as a cytoplasmic marker. Unstimulated RPTEC presented weak cytoplasmic DNase I expression but no nuclear DNase I staining (Figure 3A, first row). After stimulation of RPTEC with TNFα alone, the DNase I protein expression levels increased, DNase I translocated into the nuclei (Figure 3A, second row), and DNase I mRNA levels increased slightly (Figure 3C). After TNFα-stimulation and simultaneous transfection of the cells with DNase I mRNA-specific siRNA, DNase I mRNA expression levels were reduced to a level below that in unstimulated cells (Figure 3C), however, nuclear DNase I translocation was still weakly detectable (Figure 3A, third row). Importantly, transfection of these cells with DNase I siRNA did not affect the IL-1β mRNA levels (Figure 3C). Nuclear intensity measurement of DNase I in RPTEC cells transfected with DNase I and IL1β siRNA were represented (Figure 3B). This explains why DNase I siRNA does not interfere with endogenous IL-1β-mediated upregulation of FasR mRNA levels (Figure 3C). Thus, DNase I that translocates into the nucleus most likely derives from previously transcribed and stored DNase I protein, and its translocation is promoted by IL-1β.

Whether nuclear DNase I translocation is an IL-1 receptor-depending process was analyzed by addition of IL-1Ra to RPTEC stimulated with IL-1β. This resulted in strong reduction in IL-1β mRNA levels (Supplementary Figure 3C). However, IL-1Ra was not sufficient to eliminate the upregulation of IL-1β gene expression levels completely in response to IL-1β-stimulation in RPTEC (Supplementary Figures 3C,D) or to TNFα-stimulation (as demonstrated in Supplementary Figure 3D). This may probably explain why addition of IL-1Ra did not markedly affect mRNA levels of FasR in IL-1β- or in TNFα-stimulated cells (Supplementary Figure 3E).

**Figure 4:**
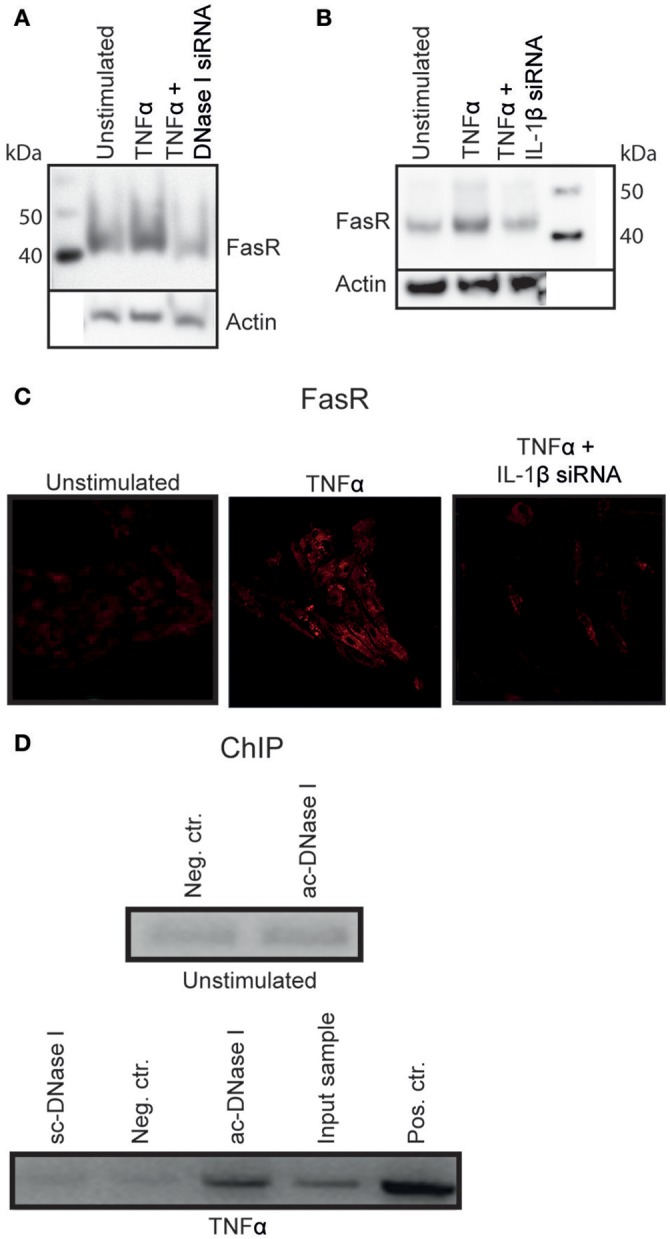
DNase I binds to the FasR promotor region. TNFα stimulated RPTEC transfected with DNase I-specific siRNA did not alter FasR protein expression severely **(A)**. Stimulation of RPTEC with TNFα and transfecting with IL-1β mRNA-specific siRNA, left the FasR protein at the same level as in unstimulated cells (**B,C**, right panel). However, stimulation of RPTEC with TNFα increased FasR expression (**B,C**, middle panel).The ChIP experiment was performed to analyse the context between nuclear DNase I translocation and upregulation of FasR **(D)**. FasR promoter-specific qPCR on sheared chromatin from unstimulated RPTEC immunoprecipitated with non-specific IgG was close to negative (Neg. ctr.), similar to sheared chromatin from unstimulated cells immunoprecipitated with the anti-DNase I IgG (Abcam (ac)-DNase I). ChIP using anti- DNase I antibody (Santa cruz (sc)-DNase I) and non-specific IgG (Neg. ctr) did not precipitate FasR-containing DNA sequences for the TNFα-stimulated cells, while the anti-DNase I antibody (ac-DNase I) precipitated DNase I in complex with FasR promoter sequences. qPCR on sheared chromatin demonstrated presence of FasR promoter DNA sequence in this sample (Input sample). As a positive control, anti-histone H3 antibody-precipitated chromatin fragments were used (D, Pos. Ctr.).

RPTEC stimulated with TNFα and transfected with DNase I specific siRNA upregulated FasR mRNA significantly (Figure 3C) in contrast to the FasR protein expression level (Figure 4A) which remained unaffected in harmony with the reduced DNase I translocation in this experiment. Importantly, prevention of nuclear DNase I translocation by IL-1β siRNA in RPTEC also reduced TNFα mediated upregulation of FasR mRNA level (Figure 3C). In addition, FasR protein expression was reduced (Figures 4B,C).

Different concentration of TNFα stimulation upregulates IL-1β and *FAS* mRNA significantly in other cell lines (Supplementary Figure 4A). In addition, nuclear DNase I translocation and its association with IL-1β mRNA levels and protein expression were also observed *in vivo* as demonstrated by data in Supplementary Figure 4. IL-1β gene and protein expression are shown in young (<6 weeks) and older mice (20 weeks) (Supplementary Figures 4A,B, respectively) and demonstrate higher IL-1β levels in 20 weeks old mice compared to young mice (Supplementary Figures 4A,B, for IL-1β mRNA and IL-1β protein *in situ*). A low amount of DNase I nuclear staining was seen in animals with low levels of renal IL-1β expression (Supplementary Figure 4C), compared to animals with higher IL-1β expression levels in which tubular cell nuclei were extensively stained (Supplementary Figure 4C, 20 weeks).

### ChIP analysis revealed that DNase I bound to the FasR promotor region

As demonstrated above, IL-1β induced nuclear DNase I translocation in TNFα-stimulated RPTEC, accompanied by upregulation of FasR mRNA and protein expression levels. Since translocated nuclear DNase I in this experiment was enzymatically inactive and its appearance in the nuclei did not result in chromatin fragmentation, (Thiyagarajan et al., 2015 see below), we hypothesized that nuclear DNase I was involved in regulation of the *FAS* gene. To test this hypothesis ChIP analyses was performed (Figure 4D) by using non-specific IgG (neg.ctr), one anti-55 kDa DNase I (Santa Cruz (sc)), and one anti-DNase I antibody specific for translocated DNase I [52 kDa DNase I, Abcam (ac)]. To determine if DNase I was associated with FasR promoter sequences in chromatin fragments precipitated by the antibodies, FasR specific qPCR was performed (Figure 4D, upper part represent data from unstimulated RPTEC and lower part demonstrate data from TNFα-stimulated RPTEC). FasR-specific qPCR on chromatin from unstimulated RPTEC immunoprecipitated with control-IgG and ac-DNase I was negative (neg.ctr). Also the anti-DNase I specific antibody from Santa Cruz (sc) did not precipitate FasR sequences. In contrast, the anti-DNase I antibody specific for translocated DNase I precipitated FasR sequences in chromatin from TNFα-stimulated RPTEC. The qPCR on purely sheared chromatin demonstrated presence of FasR DNA sequences (Input sample) and as a positive control, an anti-histone H3 antibody precipitated chromatin fragments that yielded a positive FasR-specific qPCR (pos ctr).

Thus, combining the results demonstrated that DNase I translocates into the nuclei in response to IL-1β, probably binding to the promoter region of the *FAS* gene where it may exert a function as a transcription factor for FasR.

### Apoptosis in RPTEC is induced when DNase I translocates to the nucleus and upregulates FasR in presence of exogenous FasL

**Figure 5:**
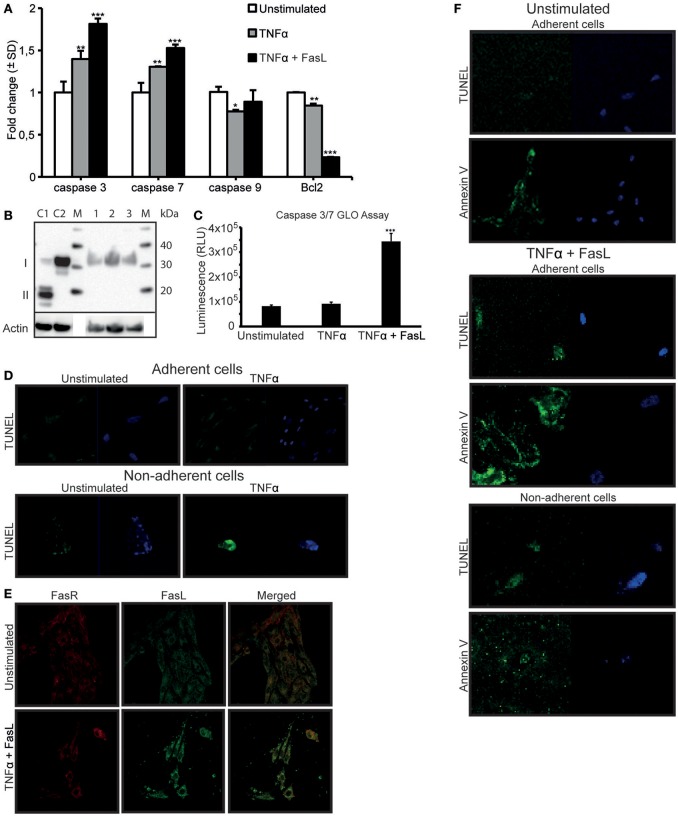
TNFα stimulation upregulates the FasR protein and caspase 3 mRNA expression levels in RPTEC, but the cells undergo apoptosis only in the presence of exogenous FasL. FasL interaction with upregulated Fas receptor (FasR) is required to induce apoptosis in RPTEC stimulated with TNFα. In TNFα-stimulated RPTEC, caspase 3 and 7 mRNA were significantly upregulated, the mRNA level of caspase 9 did not changed while Bcl2 were significantly downregulated. Additional treatment with 125 ng FasL resulted in upregulation of caspase 3 and caspase 7 mRNA levels and further downregulation of Bcl2 mRNA levels **(A)**. Notably, activated caspase 3 was not detected in unstimulated RPTEC (**B**, lane 1), RPTEC stimulated with TNFα (**B**, lane 2) as well as in RPTEC stimulated with TNFα and treated with FasL (**B**, lane 3). Controls 1 and 2 were cytochrome c treated Jurkat cells (activated caspase 3 positive, II) and untreated Jurkat cells (activated caspase 3 negative, I), lane C1 and C2 respectively. Caspase GLO 3/7 Assay were performed in RPTEC with TNFα stimulation alone and with TNFα stimulation in the presence of FasL. The results are represented from three individual experiments in relative light units (RLU) and values were subtracted from blank (media without cells) **(C)**. In adherent unstimulated or TNFα-stimulated RPTEC, no cells were TUNEL positive (**D**, upper panel). In non-adherent and unstimulated RPTEC, only very few cells could be observed and they were marginally TUNEL positive (**D**, lower panel). RPTEC stimulated with TNFα revealed a single detectable non-adherent cell that was TUNEL positive. The cells were counterstained with DAPI to ascertain their presence. As demonstrated by confocal microscopy, FasL did not bind to unstimulated RPTEC, while stimulation with TNFα resulted in strong binding of exogenously added FasL to the cell membranes (**E**, upper and lower panel, respectively). The FasL-FasR interaction induced apoptosis in RPTEC. Unstimulated adherent cells were TUNEL negative, while Annexin V stained weakly adherent unstimulated RPTEC. In this context, no non-adherent cells could be detected. When stimulating RPTEC with TNFα followed by addition of FasL, both adherent and non-adherent cells were TUNEL-positive **(F)**. Similarly, TNFα-stimulated and FasL-treated adherent and non-adherent RPTEC were strongly Annexin V-positive. The cells were counterstained with DAPI to ascertain their presence. Significances: ^*^P ≤ 0.05; ^***^P ≤ 0.0005.

In a previous study, we demonstrated that TNFα-stimulation did not directly induce apoptosis in RPTEC (Thiyagarajan et al., 2015). In light of the results described above, we analyzed a link between DNase I translocation and upregulation of FasR in absence of detectable apoptosis. In-depth analyses of several apoptotic, parameters in stimulated cells were examined. After TNFα-stimulation, a significant upregulation of FasR (Figure 1F), caspase 3 and caspase 7 mRNA levels (Figure 5A) was shown. We did not detect activated caspase 3 levels upon TNFα-stimulation (Figure 5B). However, after TNFα stimulation in the presence of 125 ng of FasL, RPTEC showed an increased caspase 3/7 activity compared to the unstimulated and TNFα stimulated cells by Caspase GLO 3/7 assay (Figure 5C).

In order to ascertain that TNFα does not induce apoptosis in RPTEC, other relevant apoptotic parameters were analyzed (Figure 5D). Since RPTEC grow as adherent cells and become non-adherent by cell death, quantification of non-adherent cells is indicative of cell death. The numbers of non-adherent RPTEC were negligible and very similar in the unstimulated or TNFα-stimulated RPTEC cultures, thus arguing against an apoptotic cell death in response to TNFα.

In an analogous approach, the TUNEL assay was negative for unstimulated and TNFα-stimulated adherent cells (Figure 5D, upper panel), while inconclusive for non-adherent cells (Figure 5D, lower panel) because of no or marginal amounts of cells in the culture medium.

Data presented so far demonstrate that TNFα-stimulation alone upregulates the FasR protein expression levels, but did not render the cells TUNEL positive. Therefore, RPTEC demonstrate a relative resistance to TNFα-mediated apoptosis, consistent with previous observations for tubular cells (Boonstra et al., 1997; Jo et al., 2002; Kunter et al., 2005). Resistance to TNFα-induced apoptosis could also be explained by the fact that RPTEC do not constitutively express FasL, which was confirmed by gene expression analysis (Supplementary Figure 1B). In order to induce apoptosis in RPTEC, we added recombinant human FasL to the cells stimulated with TNFα. As demonstrated in Figure 5E, FasL did not bind to unstimulated RPTEC, while after TNFα-stimulation; FasL bound significantly to the cell membranes and was in confocus with FasR. To identify whether the interaction of FasL with FasR induced overt apoptosis, several apoptotic markers were analyzed after TNFα- and FasL-stimulation of RPTEC. In these cells, mRNA levels of caspases 3 and 7 were significantly upregulated in contrast to the anti-apoptotic Bcl2 mRNA that was significantly reduced in presence of FasL (Figure 5A). However, as observed for TNFα-stimulated RPTEC, we could not detect activated caspase 3 in these cells after treatment with exogenous FasL by Western blot (Figure 5B). In Figure 5F, it is demonstrated that unstimulated cells were both TUNEL and Annexin V negative. However, both adherent and non-adherent TNFα-stimulated cells to which FasL was added became TUNEL-positive and Annexin-positive (Figure 5F). In this experiment, we did not observe non-adherent cells with intact morphology, rather, Annexin V stained mostly microparticle-like (Beyer and Pisetsky, 2010; Dey-Hazra et al., 2010; Dinkla et al., 2013) or blebs-like (Cocca et al., 2002) structures in the culture supernatant (Figure 5F, lower panel).

To summarize, the data demonstrate that TNFα-stimulation upregulates FasR indirectly through TNFα-mediated upregulation of IL-1β in RPTEC. IL-1β promotes nuclear translocation of DNase I, which leads to the upregulation of FasR. This cascade makes the cells highly susceptible to FasL-mediated apoptosis.

## Discussion

Data presented in this study provide new insight into regulation and function of renal DNase I. The data describe the influence of pro-inflammatory cytokines on DNase I expression, on subcellular localization and on a new and potentially important biological effect of DNase I. TNFα-stimulation of RPTEC enhanced DNase I and IL-1β mRNA and protein expression, and translocated DNase I into the nucleus with a hitherto undescribed MW of 52 kDa (Thiyagarajan et al., 2015). Stimulation of RPTEC with IL-1β translocated DNase I without enhancing its gene expression, indicating that TNFα-stimulation translocated DNase I through enhanced expression of IL-1β. Transfection of RPTEC with siRNA specific for DNase I or IL-1β mRNAs demonstrated that enhanced expression of DNase I and translocation of DNase I derived from two independent processes. Enhanced DNase I gene expression is a direct response to TNFα, independent from the effect of IL-1β, while translocation into the nucleus is a direct effect of *endogenously* expressed IL-1β. Thus, aside from demonstration that DNase I translocated into the nucleus by confocal microscopy, an even stronger argument for translocation comes from our ChIP experiment with anti-DNase I antibody. While applied to unstimulated cells, the ChIP experiment was negative for FasR promoter sequences, corresponding to the results showing that FasR expression levels was not upregulated. The same experiment using cells stimulated with TNFα demonstrated that DNase I bound to FasR promoter sequences, correlating with the results showing upregulation of FasR protein levels.

TNFα is a known inducer of apoptosis (Bradham et al., 1998; He and Ting, 2002; Schlatter et al., 2011). Nuclear translocation of DNase I as reported here could be interpreted as a part of an induced apoptotic cascade leading to apoptotic chromatin fragmentation. However, we did not observe signs of apoptosis in RPTEC upon TNFα-stimulation as demonstrated by absence of apoptotic DNA fragmentation (Thiyagarajan et al., 2015), negative TUNEL, absence of Annexin V-positive cells/blebs, and by absence of morphological changes in RPTEC corresponding to apoptosis. As has been published before, TNFα alone is not sufficient to induce apoptosis in RPTEC *in vitro* unless the cells are simultaneously treated with RNA and protein synthesis inhibitors (Kunter et al., 2005). This is explained by the fact that TNFα triggers two distinct signaling pathways leading either to apoptosis or to activation of NF-κB transcription factors, which will inhibit apoptosis through expression of anti-apoptotic genes (He and Ting, 2002; Machuca et al., 2006; Schlatter et al., 2011). In context of the present study, nuclear translocation of DNase I was not accompanied by apoptosis. This is in harmony with the fact that nuclear translocated DNase I has a MW of 52 kDa and is enzymatically inactive, while DNase I with endonuclease activity was located in the cytoplasm with a MW of 40 kDa. The 55 kDa DNase I targeted by the Santa Cruz antibody has been shown to be enzymatically inactive (Thiyagarajan et al., 2015), and located in the cytoplasm. We demonstrate a strong correlation between *de novo* expression of IL-1β, nuclear translocation of DNase I, and enhanced expression of *FAS* gene. This correlation was dependent on cytoplasmic DNase I, but also of *de novo* synthesized DNase I since cells stimulated by TNFα and transfected with DNase I siRNA demonstrated low levels of cytoplasmic 40 kDa DNase I, and weakly detectable translocated DNase I. On the other hand, stimulation of RPTEC with TNFα and transfecting cells with IL-1β siRNA resulted in increased DNase I expression, but blocked DNase I translocation. Thus, the conclusion of these results is that the nuclear translocated DNase I are directly involved in upregulation of FasR. The ChIP experiments adds strength to this assumption. Abcam anti-DNase I antibody precipitated with *FAS* promoter gene sequences. Thus, an assumed IL-1β-mediated modification of cytoplasmic DNase I into a 52 kDa protein resulted in nuclear translocation and its binding to the FasR promoter. The negative effect on this process exerted by IL-1β-specific siRNA transfection harmonizes with the conclusions that *de novo* synthesis of IL-1β accounted for the process that enhanced FasR expression. Since stimulation of cells with IL-1β directly upregulated endogenous IL-1β, this might mean that translocation of DNase I is not dependent on interaction of IL-1β with its cell membrane receptor. Rather, the direct effect of TNFα on upregulation of de novo synthesized IL-1β may indicate that IL-1β exerts its effect on DNase I translocation unlinked from interaction with its receptor. As we have shown, addition of IL-1Ra to TNFα-stimulated RPTEC did not affect mRNA level of FasR. It can be explained by an insufficient effect of IL-1Ra on IL-1β binding to its receptor, or may indicate an IL-1receptor-independent mechanism of DNase I translocation in TNFα-stimulated cells. The role of the IL-1β receptor on the processes described here is currently under investigation.

DNase I has also previously been suggested to act as a transcription factor for FasR by Oliveri et al. (Oliveri et al., 2004; Tinazzi et al., 2009). The authors demonstrated that recombinant DNase I may bind the mannose 6-phosphate receptor followed by increased surface expression of FasR. Whether the recombinant DNase I added to the cell culture was indirectly or directly involved in upregulation of the FasR was not determined. However, the data presented here may envisage the basis for the effect of DNase I in the Oliveri study. If recombinant DNase I binds directly to the mannose 6-phosphate receptor, this may enhance IL-1β production in the cells as has been demonstrated by Vidal-Vanaclocha et al. (1996). Similar to our present study, endogenous *de novo* synthesis of IL-1β in response to stimulation of the mannose 6-phosphate receptor is likely to cause translocation of endogenous DNase I into the nucleus. Thus, recombinant DNase I may drive this process, while endogenous IL-1β fulfills it by translocating endogenous DNase I to the nuclei where it may directly enhance FasR expression (studies in progress).

Our data collectively demonstrate that nuclear translocation of DNase I converts the protein from an endonuclease into a protein functioning as a transcription factor regulating the expression of the *FAS* gene. We therefore conclude that DNase I likely has a dual biological effect; DNase I is involved in processes initiating and priming the cells for apoptosis by increasing the FasR expression and function (Wajant, 2002), in addition to its well-known role in chromatin fragmentation in later phases of the apoptotic process.

## Author contributions

Conceived and designed the experiments: DT, HP, NS, KH, and OR. Performed the experiments: DT, HP, and NS. CHIP experiment performed by DT, RS, TN, and JvdV. Analyzed and interpreted the data: DT, HP, NS, LA, RS, TN, JvdV, and OR. Contributed reagents, materials, analysis tools: DT, HP, NS, LA, JvdV, and OR. Wrote the paper: DT, HP, NS, KH, LA, and OR. Revised, approved the final version of the manuscript and agreed to submit: DT, HP, NS, KH, LA, RS, TN, JvdV, and OR.

### Conflict of interest statement

The authors declare that the research was conducted in the absence of any commercial or financial relationships that could be construed as a potential conflict of interest.

## Supplementary material

Supplementary Figure 1 Expression of FasR and FasL in RPTEC cells stimulated with TNFα and IL-1β. FasR were upregulated in a dose dependent manner in RPTEC stimulated at various concentration of TNFα **(A)**. FasL mRNA is not expressed in resting RPTEC, nor induced by 20 ng/ml of TNFα stimulation for 48 h. Data are shown in comparison to mRNA expression levels of FasR **(B)**. FasL mRNA is not expressed in resting RPTEC nor induced by 2.5 ng/ml of IL-1β stimulation for 48 h **(C)**. Data are shown in comparison to mRNA expression levels of FasR. Significances: ^*^*P* < 0.05; ^***^*P* < 0.0005.

Supplementary Figure 2 RPTEC stimulated with 2.5 ng/ml of IL-1β translocates DNase I into the nucleus. The percentage of DNase I nuclear staining were calculated in both unstimulated and IL-1β stimulated cells. An average of 85% cells had stained nucleus compared to the unstimulated cells **(A)**. Various confocal images show a strong nuclear DNase I staining in cells stimulated with IL-1β compared to unstimulated cells **(B)**.

Supplementary Figure 3 FasR and IL-1β mRNA expression levels. FasR is upregulated to near maximum levels upon stimulation of RPTEC with 0.037ng/ml of IL-1β **(A)**. Stimulation of RPTEC with serial dilutions (0.037–2.5 ng/ml) of IL-1β demonstrates a dose-response relationship with endogenous IL-1β transcription **(B)**. IL-1 receptor antagonist (IL-1Ra) treatment of RPTEC stimulated with IL-1β reduces endogenous IL-1β transcription rates up to 14 times **(C)**. IL-1β stimulation of RPTEC in presence of IL-1Ra reduces IL-1β transcription, while TNFα induced IL-1β transcription was unaffected by IL-1Ra **(D)**. Since IL-1β mRNA expression was not completely lost in presence of IL-1Ra (see **C**), and as very small amounts of IL-1β (<0.05 ng/ml, see **B**) upregulate FasR expression, expression of FasR in RPTEC stimulated with IL-1β or TNFα and treated with IL-1Ra was unaffected **(E)**. Significances: ^*^*P* < 0.05; ^***^*P* < 0.0005

Supplementary Figure 4 mRNA and protein expression levels of IL-1β and *in-vivo* nuclear staining of DNase I in kidneys of pre-nephritic mice. mRNA levels of FAS and IL-1β in HEK cells (Human Embryonic Kidney cell line), LNCaP cells (Prostate cancer cell line) and MCF-7 cells (Breast cancer cell line) stimulated with 10 ng and 20 ng of TNFα **(A)**. Notably, and in harmony with the assumption that the pro-inflammatory cytokine IL-1β is involved in nuclear DNase I translocation (see text), the renal mRNA **(B)** and protein **(C)** level of IL-1β was higher in kidneys with nuclear DNase I than in kidneys with DNase I predominantly detected in the cytoplasm **(D)**. Significances: ^*^*P* ≤ 0.05; ^**^*P* ≤ 0.005; ^***^*P* ≤ 0.001.
