# Supplementary figures and images for: IL-1β Promotes a New Function of DNase I as a Transcription Factor for the Fas Receptor Gene
Source: Front Cell Dev Biol. 2018 Feb 6;6:7. doi: 10.3389/fcell.2018.00007 (PMC5807897; doi:10.3389/fcell.2018.00007)

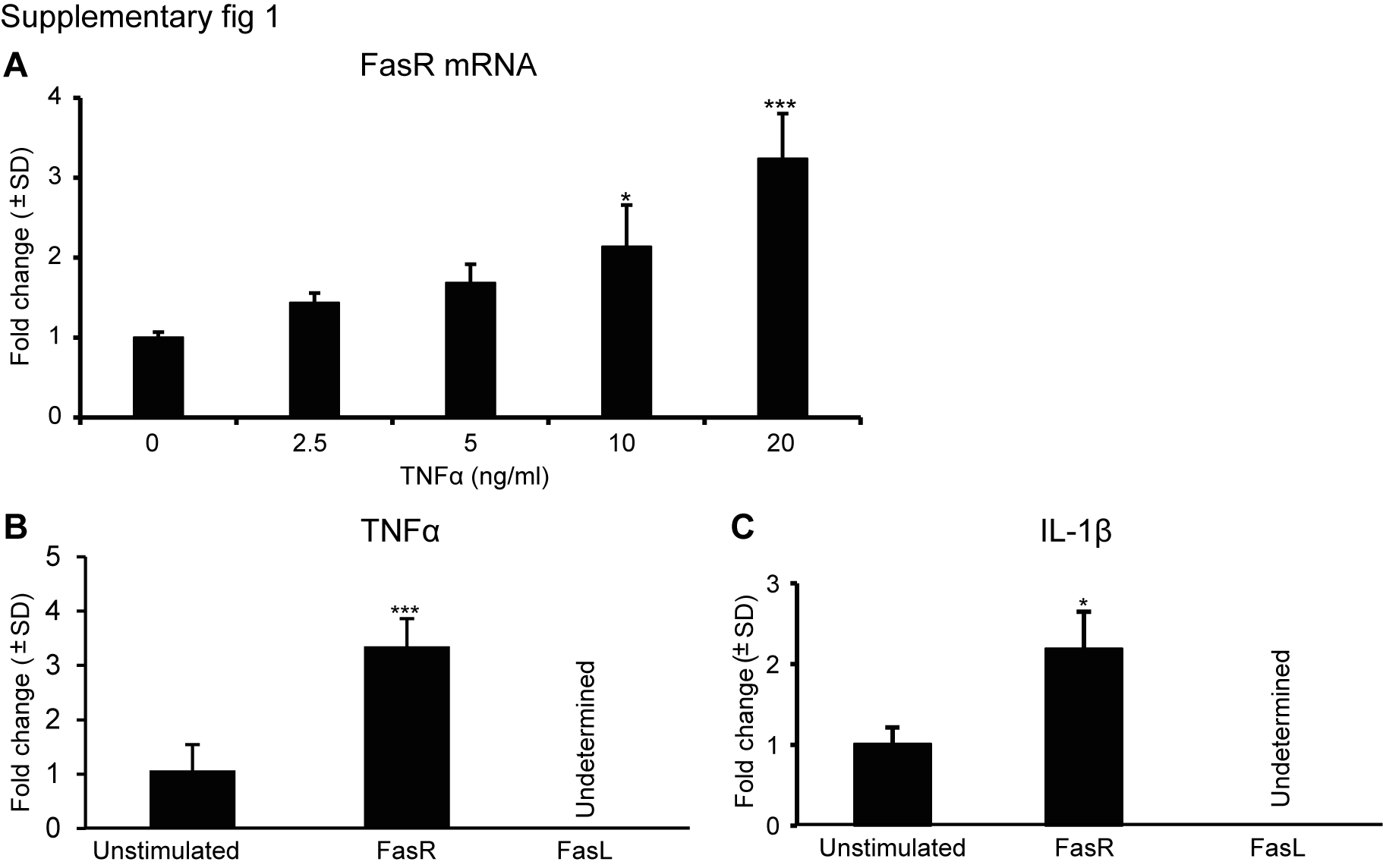

Supplement: Supplementary Figure 1 — Expression of FasR and FasL in RPTEC cells stimulated with TNFα and IL-1β. FasR were upregulated in a dose dependent manner in RPTEC stimulated at various concentration of TNFα (A). FasL mRNA is not expressed in resting RPTEC, nor induced by 20 ng/ml of TNFα stimulation for 48 h. Data are shown in comparison to mRNA expression levels of FasR (B). FasL mRNA is not expressed in resting RPTEC nor induced by 2.5 ng/ml of IL-1β stimulation for 48 h (C). Data are shown in comparison to mRNA expression levels of FasR. Significances: *P < 0.05; ***P < 0.0005. [file Image1.TIF]

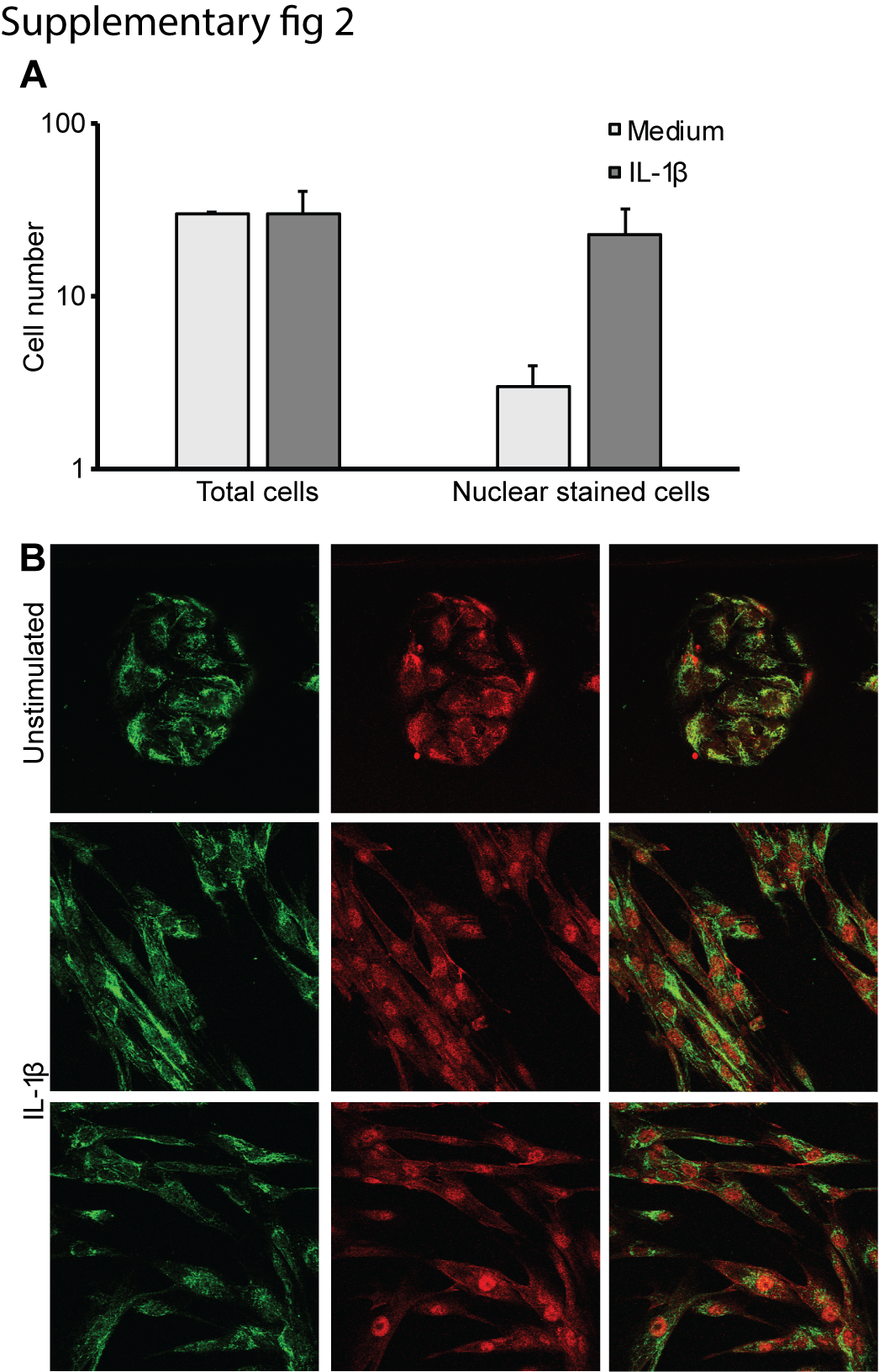

Supplement: Supplementary Figure 2 — RPTEC stimulated with 2.5 ng/ml of IL-1β translocates DNase I into the nucleus. The percentage of DNase I nuclear staining were calculated in both unstimulated and IL-1β stimulated cells. An average of 85% cells had stained nucleus compared to the unstimulated cells (A). Various confocal images show a strong nuclear DNase I staining in cells stimulated with IL-1β compared to unstimulated cells (B). [file Image2.TIF]

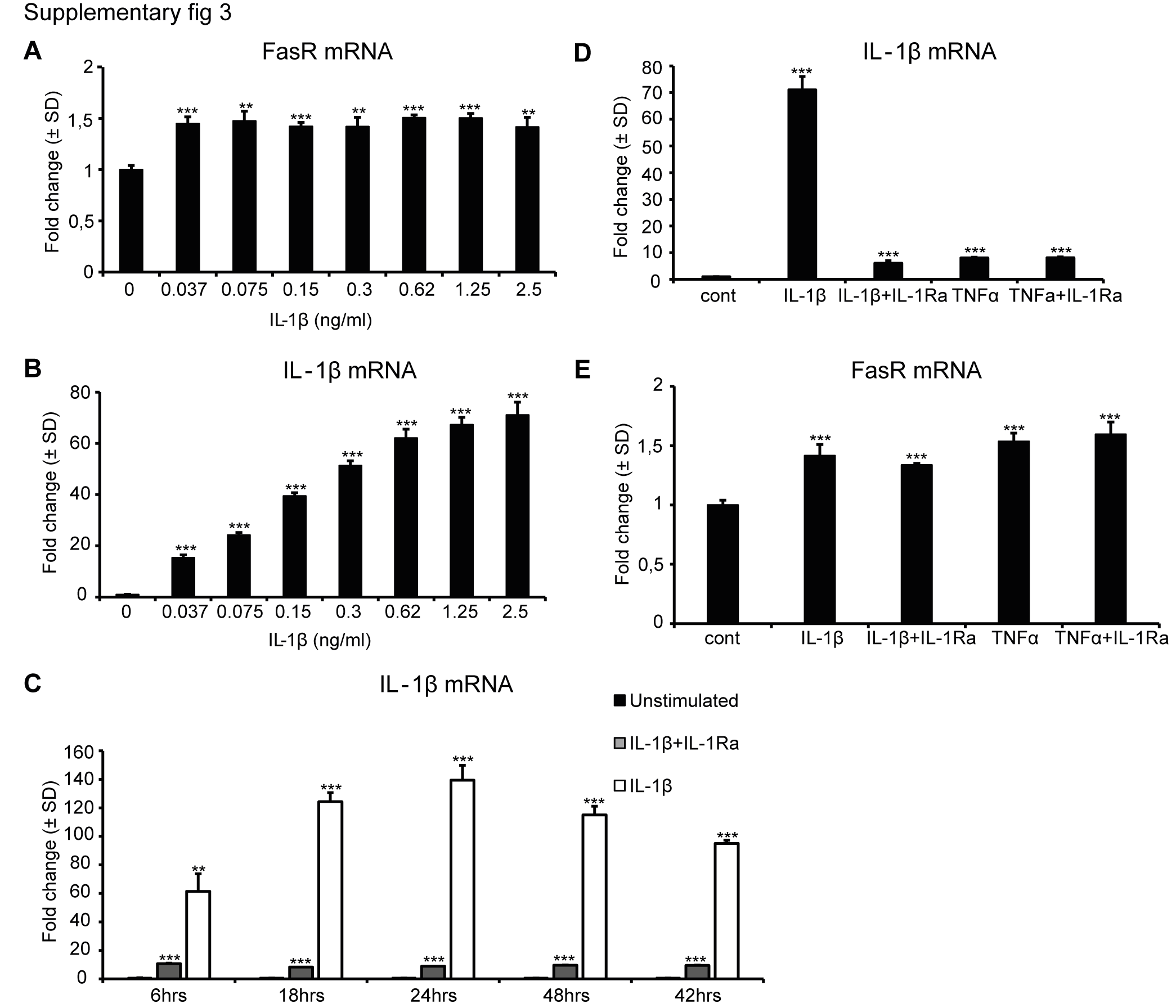

Supplement: Supplementary Figure 3 — FasR and IL-1β mRNA expression levels. FasR is upregulated to near maximum levels upon stimulation of RPTEC with 0.037ng/ml of IL-1β (A). Stimulation of RPTEC with serial dilutions (0.037–2.5 ng/ml) of IL-1β demonstrates a dose-response relationship with endogenous IL-1β transcription (B). IL-1 receptor antagonist (IL-1Ra) treatment of RPTEC stimulated with IL-1β reduces endogenous IL-1β transcription rates up to 14 times (C). IL-1β stimulation of RPTEC in presence of IL-1Ra reduces IL-1β transcription, while TNFα induced IL-1β transcription was unaffected by IL-1Ra (D). Since IL-1β mRNA expression was not completely lost in presence of IL-1Ra (see C), and as very small amounts of IL-1β (<0.05 ng/ml, see B) upregulate FasR expression, expression of FasR in RPTEC stimulated with IL-1β or TNFα and treated with IL-1Ra was unaffected (E). Significances: *P < 0.05; ***P < 0.0005 [file Image3.TIF]

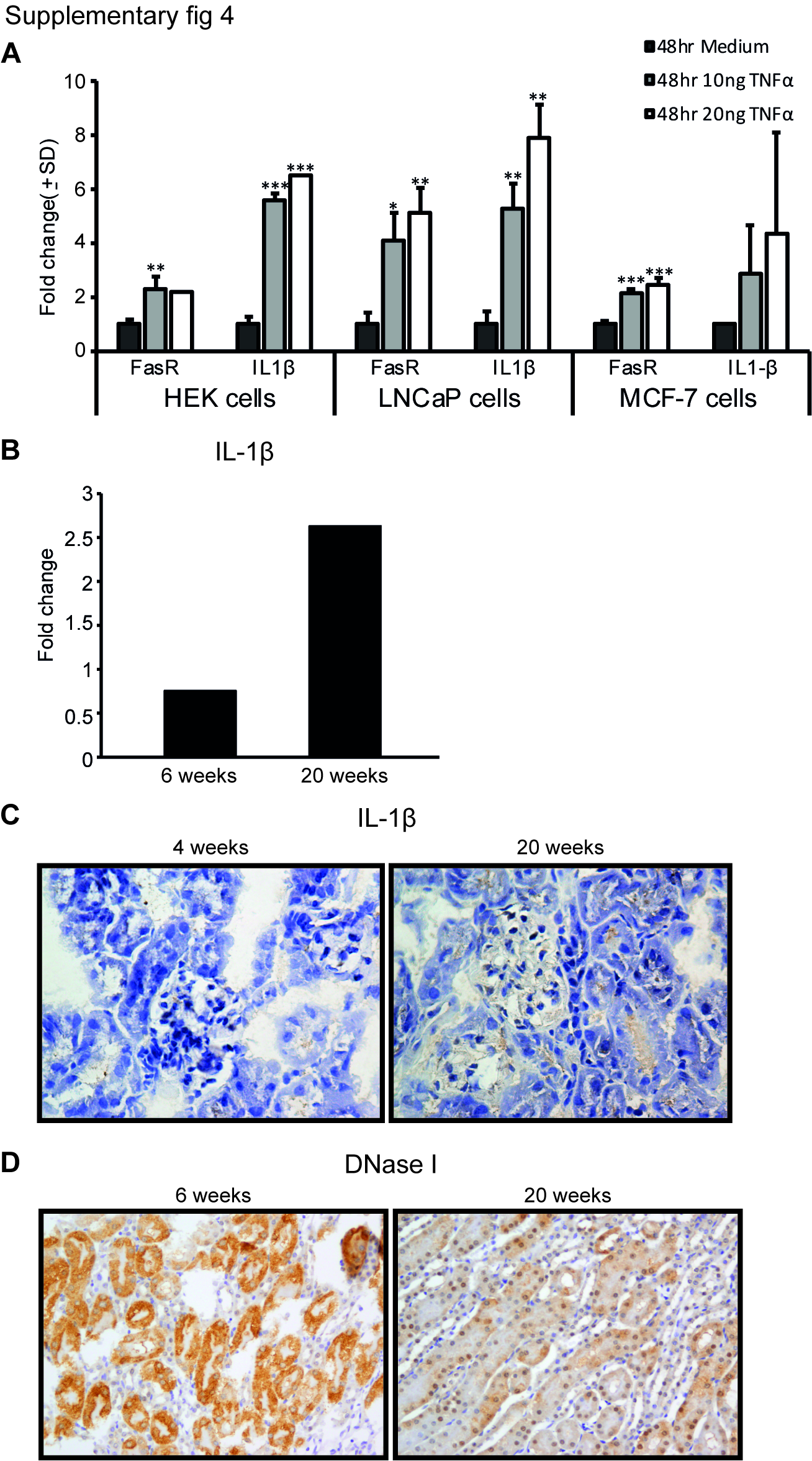

Supplement: Supplementary Figure 4 — mRNA and protein expression levels of IL-1β and in-vivo nuclear staining of DNase I in kidneys of pre-nephritic mice. mRNA levels of FAS and IL-1β in HEK cells (Human Embryonic Kidney cell line), LNCaP cells (Prostate cancer cell line) and MCF-7 cells (Breast cancer cell line) stimulated with 10 ng and 20 ng of TNFα (A). Notably, and in harmony with the assumption that the pro-inflammatory cytokine IL-1β is involved in nuclear DNase I translocation (see text), the renal mRNA (B) and protein (C) level of IL-1β was higher in kidneys with nuclear DNase I than in kidneys with DNase I predominantly detected in the cytoplasm (D). Significances: *P ≤ 0.05; **P ≤ 0.005; ***P ≤ 0.001. [file Image4.TIF]
